# A Reductionist Approach Using Primary and Metastatic Cell–Derived Extracellular Vesicles Reveals Hub Proteins Associated with Oral Cancer Prognosis

**DOI:** 10.1016/j.mcpro.2021.100118

**Published:** 2021-06-27

**Authors:** Ariane Fidelis Busso-Lopes, Carolina Moretto Carnielli, Flavia Vischi Winck, Fábio Malta de Sá Patroni, Ana Karina Oliveira, Daniela Campos Granato, Rute Alves Pereira e Costa, Romênia Ramos Domingues, Bianca Alves Pauletti, Diego Mauricio Riaño-Pachón, Juliana Aricetti, Camila Caldana, Edgard Graner, Ricardo Della Coletta, Kelly Dryden, Jay William Fox, Adriana Franco Paes Leme

**Affiliations:** 1 Laboratório Nacional de Biociências - LNBio, Centro Nacional de Pesquisa em Energia e Materiais - CNPEM, Campinas, SP, Brazil; 2 Laboratório de Biologia de Sistemas Regulatórios, Departamento de Bioquímica, Instituto de Química, Universidade de São Paulo, São Paulo, SP, Brazil; 3 Laboratório Nacional de Biorrenováveis - LNBR, Centro Nacional de Pesquisa em Energia e Materiais - CNPEM, Campinas, SP, Brazil; 4 Max Planck Institute of Molecular Plant Physiology, Am Mühlenberg, Potsdam-Golm, Germany; 5 Departamento de Diagnóstico Oral, Faculdade de Odontologia de Piracicaba, Universidade Estadual de Campinas, Piracicaba, SP, Brazil; 6 Molecular Electron Microscopy Core, University of Virginia, Charlottesville, Virginia, USA; 7 Department of Microbiology, Immunology and Cancer Biology, University of Virginia, Charlottesville, Virginia, USA

**Keywords:** extracellular vesicles, mouth neoplasms, oral squamous cell carcinoma, lymph node metastasis, proteomics, mass spectrometry, multi-omics, integrative analysis, prognosis, AUC, area under the curve, EV, extracellular vesicle, GO, Gene Ontology, HNCDB, Head and Neck Cancer Database, HNSCC, head and neck squamous cell carcinoma, KEGG, Kyoto Encyclopedia of Genes and Genomes, LMPD, LIPID MAPS Proteome Database, NTA, nanoparticle tracking analysis, OSCC, oral squamous cell carcinoma, PC, phosphatidylcholine, PCA, principal component analysis, PE, phosphatidylethanolamine, ROC, receiver operating characteristic, TCGA, The Cancer Genome Atlas, THCA, thyroid cancer

## Abstract

Oral squamous cell carcinoma (OSCC) has high mortality rates that are largely associated with lymph node metastasis. However, the molecular mechanisms that drive OSCC metastasis are unknown. Extracellular vesicles (EVs) are membrane-bound particles that play a role in intercellular communication and impact cancer development and progression. Thus, profiling EVs would be of great significance to decipher their role in OSCC metastasis. For that purpose, we used a reductionist approach to map the proteomic, miRNA, metabolomic, and lipidomic profiles of EVs derived from human primary tumor (SCC-9) cells and matched lymph node metastatic (LN1) cells. Distinct omics profiles were associated with the metastatic phenotype, including 670 proteins, 217 miRNAs, 26 metabolites, and 63 lipids differentially abundant between LN1 cell– and SCC-9 cell–derived EVs. A multi-omics integration identified 11 ‘hub proteins’ significantly decreased at the metastatic site compared with primary tumor–derived EVs. We confirmed the validity of these findings with analysis of data from multiple public databases and found that low abundance of seven ‘hub proteins’ in EVs from metastatic lymph nodes (ALDH7A1, CAD, CANT1, GOT1, MTHFD1, PYGB, and SARS) is correlated with reduced survival and tumor aggressiveness in patients with cancer. In summary, this multi-omics approach identified proteins transported by EVs that are associated with metastasis and which may potentially serve as prognostic markers in OSCC.

## Highlights

• Proteomic, miRNA, metabolomic, and lipidomic profiles were mapped in oral cancer EVs.
• The molecular profile of EVs was associated with the lymph node metastatic phenotype.
• A multi-omics integrative analysis revealed 11 highly connected ‘hub proteins.’
• ‘Hub proteins’ from EVs are candidates as prognostic markers in oral cancer.

## Graphical Abstract

**Figure:**
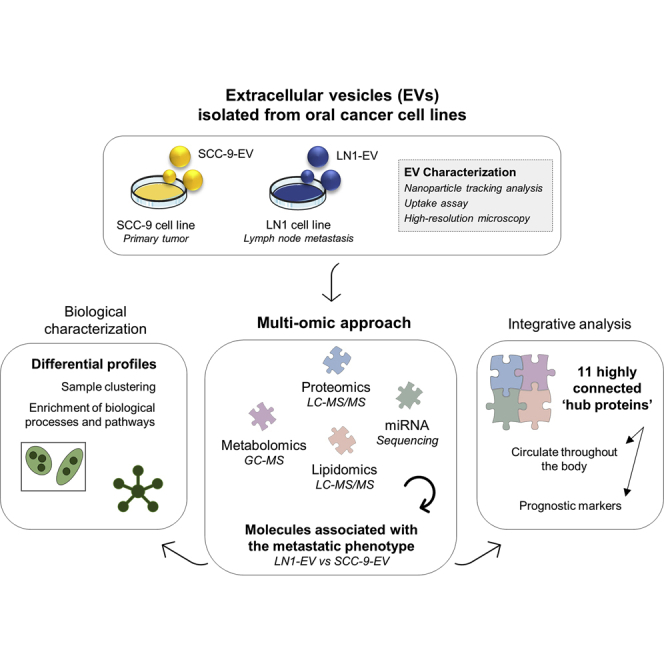

## In Brief

A multi-omics strategy was used to map the proteome, miRNA, metabolome, and lipidome of EVs derived from human primary tumor (SCC-9) cells and matched lymph node metastatic (LN1) cells. Differentially abundant molecules associated with the metastatic phenotype were enriched for key processes and pathways. An integrative analysis revealed 11 ‘hub proteins’ that are correlated with reduced survival and tumor aggressiveness in patients with cancer according to public databases. These EV molecules are candidates as prognostic markers in oral cancer.

Head and neck squamous cell carcinoma (HNSCC) exhibits high incidence and morbidity, and oral squamous cell carcinoma (OSCC) comprises over 90% of all cases, showing a 5-year survival rate of 50% (1). The presence of lymph node metastasis in the neck remains a major prognostic factor affecting patients with OSCC, which can decrease the 5-year survival rates to lower than 50% (2). Thus, a deep profiling of the molecular events in OSCC is crucial to understand disease aggressiveness and identify additional parameters or molecular markers that assist in determining patient outcome.

Extracellular vesicles (EVs) are important mediators in the intercellular communication and in several physiological processes (3). In fact, EVs comprise all types of secreted membrane vesicles and can be divided into exosomes, which are small membrane vesicles from endosome origin, 30 to 100 nm in diameter, and microvesicles, which are plasma membrane derived with a size range of 50 nm to 1000 nm (4, 5). EVs harbor a specific subset of proteins, miRNA, sRNA, mRNA, DNA, metabolites, and lipids, and their content is defined to deliver specific messages to the recipient cells (3, 5, 6). Different cell types, including cancer cells, can actively release vesicles to the extracellular environment (7, 8), and increasing evidence indicates that EVs are mediators of cancer development, progression, and metastasis (7, 9, 10). Thus, EVs hold great promise for the discovery of novel biomarkers for clinical diagnosis and monitoring of cancer (11, 12).

The promotion of tumor growth and progression by tumor cell–derived exosomes has been demonstrated in mouse models. It was evidenced that the control of metastatic progression may occur through the crosstalk between tumor-derived exosomes and bone marrow progenitor cells (10). In addition, tumor-derived EVs are important factors to determine the future sites of metastasis by mediating the development of premetastatic niches, a condition where the microenvironment is prepared for the colonization of circulating tumor cells. This was demonstrated for pancreatic ductal adenocarcinomas–derived exosomes, which induced liver premetastatic niche formation (9), as well as in a study showing that exosomes from different tumor cells present a predicted destination through the presence of integrins inserted in the membrane (13), highlighting tumor-derived exosomes as important factors in organ-specific metastasis. Thus, targeting tumor-secreted vesicles has emerged as a potential tool for the detection and understanding of human cancer progression, including head and neck tumors.

An increasing number of vesicular components have been identified in the last years, including proteins, mRNAs, miRNAs, and lipids (14). Therefore, screening of EVs by different omics approaches represent a rich strategy to help deciphering the vesicular biology and its role in a specific disease. Omics techniques have been broadly used to determine the complexity of biological systems and uncover the molecular signatures underlying the cellular phenotypes (15). The integrative approach of multi-omics data may enhance the understanding of the molecular dynamics involved in the pathophysiology of diseases and may lead to novel strategies for early detection, prevention, and treatment in cancer (16, 17, 18, 19). In the present study, we examined the molecular repertoire of EVs released by primary site tumor cells (SCC-9) and their paired lymph node metastatic (LN1) cells to explore how EVs play a significant role in OSCC dissemination and lymph node metastasis. Using high-throughput multi-omics techniques and data-integration strategies, we determined the proteomic, miRNA, metabolomic, and lipidomic profiles of SCC-9- and LN1-derived EVs and correlated with transcript levels and prognosis using public databases. Our results indicate that OSCC-derived EVs carry specific cargoes associated with the metastatic phenotype. We also revealed a set of central molecules, the ‘hub proteins’, that may be secreted in blood and are candidates as prognostic markers in patients with oral cancer.

## Experimental Procedures

### Cell Line and Culture Conditions

Human OSCC cell line SCC-9 (ATCC CRL1629), human foreskin fibroblast cell line BJ-5ta, and human umbilical vein endothelial cell line HUVEC were obtained from the American Type Culture Collection. The SCC9-LN1 (LN1) cell line is derived from metastasized cells collected from primary lymph nodes of animals that received SCC-9 cells (20). A primary human oral fibroblast cell line was established using tissue explants as described previously (21). SCC-9 and LN1 cells were cultured in Dulbecco's modified Eagle's medium (DMEM) HAM's F12 media, HUVEC and primary fibroblasts were maintained in DMEM, and BJ-5ta was cultured in DMEM 199. All the media contained 10% fetal bovine serum and antibiotics, such as penicillin (100 mg/L) and streptomycin (100 mg/L), and were supplemented with hydrocortisone (100 mg/L).

### EV Isolation

EVs were isolated through differential centrifugation based on previous methodology (22). Briefly, SCC-9 and LN1 cells were cultured until 80% cell confluence in 150-mm-diameter plates, washed three times with PBS, and further cultivated for 48 h in media without fetal bovine serum, at 37 °C and 5% CO_2_. After serum deprivation treatment, the conditioned media (200 ml) was collected and centrifuged at 200*g* for 5 min, 2000*g* for 15 min, 3500*g* for 30 min, and 10,000*g* for 90 min. The cleared supernatant was further ultracentrifuged at 100,000*g* for 90 min at 4 °C, and vesicle-containing pellets were washed with PBS by ultracentrifugation for 1.5 h at the same speed. The samples were stored at −80 °C until further use.

### Immunolabeling of EVs

EVs isolated from SCC-9 and LN1 cells (1 × 10e10 particles) were incubated in 400 μl of the blocking solution (1% bovine serum albumin in PBS) for 2 h at 4 °C. The primary antibody against protein Annexin-2 (BD Biosciences) or Flotillin-1 (Sigma) was added in the concentration of 1:200 directly in the blocking solution containing the EVs and incubated at room temperature (RT) for 1 h with gentle agitation. The suspension of EVs was further cleaned by eluting the suspension by centrifugation (50*g*, 30 s, 22 °C) through a mini spin column containing Sephadex G-10 resin (50-mg dry weight prereconstituted in PBS) pre-equilibrated for 2 h at 4 °C with the blocking solution. The secondary antibody Alexa Fluor 568 (1:1000) (Life Technologies) was added to the eluate, and the suspension was incubated for 1 h at RT with gentle agitation in the dark. The EV suspension was again cleaned as mentioned above using Sephadex G10, and the final eluate was analyzed directly through fluorescence nanoparticle tracking analysis (NTA).

### NTA

NTA was performed using a NanoSight NS300 instrument (NanoSight), equipped with a green laser illumination (532 nm laser). Aliquots of the isolated EVs were diluted 500 times and measured at 18 °C in the PBS solution for 60 s with gain adjustments. Data capture and analysis were performed with the software NTA 2.3 Build 0013 with equal parameters for the internal comparisons of nonfluorescence and fluorescence profiles, except that camera gain was set to the maximum value for the acquisition of fluorescence NTA profiles. For fluorescence NTA, a 565-nm-long pass filter was used for specific detection of immunolabeled EVs.

### Transmission Electron Microscopy

For transmission electron microscopy, EVs from SCC-9 and LN1 cell lines were resuspended in PBS (2.5 × 10e8 particles per grid) and adsorbed on an Ultrathin Carbon Film/Haley Carbon 400 mesh copper grid positively charged with 15 mA for 25 s. The carbon grids were stained with 2% uranyl acetate and analyzed in the transmission electron microscope JEOL 1400 PLUS (JEOL Ltd) equipped with a tungsten filament and operated at an acceleration voltage of 120 kV. The images were acquired in an OneView camera (4K × 4K pixel) using the Software Gatan DigitalMicrograph (Gatan Inc).

### Cryo-EM

EVs from SCC-9 and LN1 cell lines (1 × 10e12 particles) were vitrified by standard methods for cryo-EM. An aliquot was applied to a glow-discharged, perforated carbon-coated grid (2/2-3C C-flats), blotted with filter paper, and rapidly plunged into liquid ethane. Low-dose images were recorded on an FEI Tecnai F20 Twin Transmission Electron Microscope (FEI) operating at 120 kV, at a magnification of 29,000× or 62,000× with a pixel size of 0.37 nm or 0.18 nm, respectively, at the specimen level, and at a nominal under focus ranging from 1 to 4 μm. All images were recorded with a Gatan 4K × 4K pixel CCD camera. The grids were stored in liquid nitrogen and then maintained in the microscope at −180 °C using a Gatan 626 cryo-stage.

### EV Uptake

Cellular uptake of SCC-9 EVs and LN1 EVs was analyzed using the Operetta High Content Imaging System (PerkinElmer). HUVEC, BJ-5ta, and primary fibroblast cell lines were used as recipient cells. EVs (6 × 10e7 particles) were labeled with 2 μM 1,1′-dioctadecyl-3,3,3′,3′-tetramethylindodicarbocyanine, 4-chlorobenzenesulfonate salt (emission at 660 nm) (Molecular Probes–Thermo Scientific) and incubated with recipient cells (1 × 10e4) for 48 h at 37 °C and 5% CO_2_. The cells were fixed with 4% paraformaldehyde for 20 min at RT and labeled using 4′,6-diamidino-2-phenylindole (emission at 488 nm) (Thermo Scientific) and CellTracker green CMFDA (emission at 517 nm; Thermo Scientific) before image acquisition.

### Proteomics Analysis of EVs and Cells

SCC-9 and LN1 cells (three processing replicates for each group) and EVs (3 × 10e10 particles; three processing replicates for each group; three technical replicates for each processing replicate) were submitted to in-gel digestion with trypsin (23). Peptide desalting was performed using StageTips method in C18 Empore disks (3M) (24). Peptides from cell samples were quantified using the Pierce Quantitative Colorimetric Peptide Assay (Thermo Scientific), and 2 μg was submitted to subsequent analysis. The samples were analyzed by LC-MS/MS on an ETD enabled Orbitrap Velos mass spectrometer (Thermo Fisher Scientific) connected to the EASY-nLC system (Proxeon Biosystems) through a Proxeon nanoelectrospray ion source. Peptides were separated by a 2 to 90% acetonitrile gradient in 0.1% formic acid using an analytical column EASY-Column (10 cm × id 75 μm, 3-μm particle size) at a flow rate of 300 nl/min over 80 min. The nanoelectrospray voltage was set to 2.2 kV, and the source temperature was 275 °C. All instrument methods were set up in the data-dependent acquisition mode. The full-scan MS spectra (m/z 300–2000) were acquired in the Orbitrap analyzer after accumulation to a target value of 1e6. Resolution in the Orbitrap was set to r = 60,000, and the 20 most intense peptide ions with charge states ≥2 were sequentially isolated to a target value of 5000 and fragmented in the linear ion trap by low-energy CID (normalized collision energy of 35%). The signal threshold for triggering an MS/MS event was set to 1000 counts. Dynamic exclusion was enabled with an exclusion size list of 500, exclusion duration of 60 s, and repeat count of 1. An activation q = 0.25 and activation time of 10 ms were used. Identification of proteins was performed with MaxQuant v.1.5.8.0 (25, 26) against the UniProt Human Protein Database (92,180 protein sequences, 36,693,332 residues, released March 2016) using the Andromeda search engine. Carbamidomethylation was set as fixed modification and N-terminal acetylation and oxidation of methionine as variable modifications; maximum two trypsin missed cleavage and a tolerance of 4.5 ppm for precursor mass and 0.5 Da for fragment ions were set for protein identification. A maximum of a 1% false discovery rate was set for both protein and peptide identification. Protein quantification was performed using the label-free quantitation algorithm implemented in MaxQuant software, reflecting a normalized protein quantity deduced from all peptide intensity values. A minimal ratio count of 1 and a 2-min window for matching between runs were required for quantitation. Proteoforms were automatically merged in a single protein group, except when identified by at least one unique peptide. Protein identifications assigned as ‘Reverse’ and ‘Only identified by site’ were excluded from further analysis. Contaminants were not removed from the dataset because keratins are of special interest in the study of squamous cells. The raw data obtained for cells and EVs were run independently and combined in a unique MaxQuant search to further compare the proteomes from both sources. Label-free quantitation intensity values were used for statistical tests.

### miRNA Sequencing of EVs

Total RNA was extracted from SCC-9- and LN1-isolated EVs using TRIzol reagent, according to manufacturer’s instructions (Life Technologies). Quality was accessed by Bioanalyzer 2100 instrument with the Agilent RNA 6000 Nano kit (Agilent Technologies Inc). Small RNA sequencing was performed by the Macrogen Inc, through the construction of six libraries (three processing replicates from SCC-9 EVs and three processing replicates from LN1 EVs) and sequencing of 50-nt single-end reads, with generation of approximate 2.5 million reads per sample on average using Illumina MiSeq instrument (Illumina). The sequencing data were extracted from FASTQ files and processed using miRDeep v. 2.0.0.7 (27). Illumina adapter sequences were trimmed using Trimmomatic (28), and reads with size ranging from 15 to 31 nucleotides in length were kept for mapping purposes. Mature and hairping miRNA sequence reads from *Homo sapiens* were obtained from the miRBase database (http://www.mirbase.org/) and used for mapping purposes and miRNA identification.

### Metabolomics Analysis of EVs

Primary metabolites were extracted from SCC-9- and LN1-derived EVs (5 × 10e10 particles; five processing replicates for each group) using methyl-tert-butyl-ether extraction buffer (29). Internal standards (C13 sorbitol) were spiked in the extraction buffer for assessing extraction performance and metabolite recovery. Polar fractions were concentrated, derivatized with N-methyl-N-trimethylsilyltrifluoroacetamide, and analyzed by GC (7890 N, 210 Agilent) coupled to TOF-MS (Pegasus HT, LECO) in both split (1:15 and 1:50) and splitless modes (30). Processing replicates, quality controls (*i.e.*, mix of chemical standards), and blank samples were randomized and included in the running queue. Chromatograms were exported from LECO ChromaTOF software (version 3.25) to R v3.2.2 (https://www.r-project.org) for subsequent analysis. Peak detection, retention time alignment based on FAMEs, and mass spectral comparison with an in-house reference library were performed using TargetSearch (31). Metabolite identification was also manually supervised. Metabolites were quantified based on the peak intensity for a selected mass and subsequently normalized to total ion count.

### Lipidomics Analysis of EVs

Lipidomic profiles were determined in EV samples from SCC-9 and LN1 cell lines (1 × 10e10 particles; three processing replicates for each group) by metaSysX company (Germany). Sample preparation was performed according to metaSysX standard procedure (29). The samples were measured with an ACQUITY Reversed-Phase Ultra Performance Liquid Chromatography (Waters) coupled to a Q Exactive mass spectrometer (Thermo Fisher Scientific). Chromatograms were recorded in full-scan MS-positive and MS-negative modes (mass range 100–1500 m/z) as well as in dd-MS2 top three mode (data-dependent MS) with the following settings: full-scan MS mode (mass range 100–1500 m/z) and normalized collision energy 25 for the identification of the fatty acid composition. Extraction of the LC-MS data was accomplished with the software Refiner MS 10.5 (Genedata, http://www.genedata.com). Alignment and filtration of the LC-MS data were completed using an in-house software. The annotation of the content of the samples was accomplished by matching the extracted data from the chromatograms with a library of reference compounds in terms of accurate mass and retention time. In addition, the fatty acid composition was accessed using an in-house–developed algorithm.

### Statistical Analysis and Functional Characterization

Proteins, miRNA, metabolites, and lipids data were log_2_ transformed and used to determine differentially abundant molecules between LN-1- and SCC9-derived EVs and/or cells in Perseus v. 1.3.0.4 software (Student's *t* test; *p*-value ≤ 0.05) (32). SCC-9 EVs and LN1 EVs were grouped according to molecular profiles using principal component analysis (PCA) in the web server MetaboAnalyst 4.0 (33), and hierarchical cluster heat maps in R environment. The overlay between molecules for each condition was visualized in Venn diagrams generated in FunRich tool (34). miRNA target gene prediction was done for differentially expressed miRNAs using the miRNet platform (35), which contains experimentally validated miRNA-target interactions from ten databases (miRTarBase, TarBase, miRecords, SM2miR, Pharmaco-miR, miR2Disease, PhenomiR, StarBase, EpimiR, miRDB). Kyoto Encyclopedia of Genes and Genomes (KEGG) database (36) was used to map overrepresented pathways among the miRNA-targeted mRNAs. Meaningful Gene Ontology (GO) biological processes significantly enriched in proteomics and metabolomics data were determined in FunRich (34) and MetaboAnalyst 4.0 (37) tools, respectively. The subcellular location for proteomics dataset was assigned from information available in the Human Protein Atlas (38). A *p*-value ≤ 0.05 was used to determine significance in functional characterization analysis, when feasible.

### Integrative Analysis of Multi-Omics Data

To indicate OSCC-derived EVs molecules that are highly connected with other molecules identified across the omics datasets, we developed an integrative approach based on physical or functional associations using Python programing language. First, proteins, miRNA, and metabolites differentially abundant between LN1 EVs and SCC-9 EVs (LN1 EVs *versus* SCC-9 EVs, Student's *t* test, *p*-value ≤ 0.05) were selected for data integration considering physical associations. miRNA-target experimentally validated interactions were assigned from miRNet tool (35) and merged with miRNA and proteins differentially abundant between LN1- and SCC-9-derived EVs from our dataset. Differential metabolites were included in the workflow considering gene and metabolic compounds interaction retrieved from KEGG reactions (36). Finally, to identify molecules functionally associated with lipids, differential proteins (LN1 EVs *versus* SCC-9 EVs, Student's *t* test, *p*-value ≤ 0.05) were searched against the LIPID MAPS Proteome Database (LMPD), which retrieves proteins involved in lipid processes and pathways (39). The final network was visualized on Cytoscape 3.4.0 (40). Node sizes were used to represent fold change values for each dataset, and geometric shapes showed different omics data types. Central proteins interacting with the other omics molecules were named ‘hub proteins.’ The correlation of mean intensities between LN1 and SCC-9 cells and EVs for ‘hub proteins’ was evaluated using Pearson correlation coefficient (r). The proteins were submitted to GO annotation of significantly overrepresented biological processes using Molecular Signatures Database from Gene Set Enrichment Analysis software (*p*-value ≤ 0.05) (41, 42).

### Search for Prognostic Markers Using Public Databases

Single ‘hub proteins’ assigned in the multi-omics integrative analysis were associated with clinical and pathological features from patients with cancer using the public databases The Cancer Genome Atlas (TCGA), GSE41613, E-MTAB-1328, and GSE65858. First, transcript levels were retrieved from the public repository TCGA available in the Genomic Data Commons Data Portal (https://portal.gdc.cancer.gov) for oral and other cancer types (43, 44). The association with clinical and pathological features was performed using gene expression information from primary tumors and clinical data retrieved from patients with OSCC in TCGA repository, totalizing 331 patients included from the following oral areas: the (i) alveolar ridge, (ii) base of the tongue, (iii) buccal mucosa, (iv) floor of the mouth, (v) hard palate, (vi) oral cavity, and (v) oral tongue. The selected targets were evaluated according to different clinical categories, as follows: (i) recurrence, (ii) death status, (iii) lymph and vascular invasion, (iv) margin status, (v) tumor histological grade, (vi) lymph node status, (vii) stage, (viii) tumor size, (ix) perineural invasion, and (x) extracapsular nodal spread. For unbiased group assignment, we used *mclust* package (45) under R environment. Data were tested for normality and homogeneity of variance using the Shapiro–Wilk test (*p*-value ≤ 0.05) to drive decisions of parametric or nonparametric tests for group comparison with the clinical categories (46). The power of proteins to discriminate patients according to clinical features was evaluated by the construction of receiver operating characteristic (ROC) curves using Random Forest. The area under the curve (AUC) was measured, and the decision threshold was assigned to the value 70%. We also evaluated if the selected proteins were associated with metastasis (primary site tissue *versus* metastasis tissue), considering gene expression profiles for multiple cancer types with information available in TCGA for both primary tumor and metastasis with more than three samples per site, as follows: skin cutaneous melanoma (primary site: 103 samples; metastasis: 367 samples), THCA (thyroid cancer, 502; 8), and breast invasive carcinoma (1.102; 7).

Next, the Head and Neck Cancer Database (HNCDB) (47) and PROGgeneV2 (48) were used to create survival plots based on gene expression of input genes in the GSE41613 (49), E-MTAB-1328 (50), and GSE65858 (51) datasets, which contains clinical and molecular information from 167 patients with OSCC, 89 patients with HNSCC, and 269 patients with HNSCC, respectively. Median gene expression was used as a cut-off to determine low and high expressions of selected markers, and *p*-values were obtained by log-rank tests for Kaplan–Meier curves. We also associated TCGA transcript information for selected proteins with survival data in multiple tumor types using the Human Protein Atlas platform (52). A *p*-value ≤ 0.05 was used to determine significance.

### Search for Circulating Molecules in Blood Using a Public Repository

The presence of proteins from our multi-omics approach in blood samples was determined using The Human Protein Atlas (http://www.proteinatlas.org) repository. A list of proteins and the estimated concentrations in human plasma from healthy donors based on MS-based proteomics was downloaded from The Human Plasma Proteome available in the Human Protein Atlas platform (downloaded on February 20, 2020; https://www.proteinatlas.org/humanproteome/blood) and used to estimate the presence and abundance of our proteins of interest on this biological fluid.

### Experimental Design and Statistical Rationale

Herein, we determined the role of multiple molecules carried by primary tumor (SCC-9)- and lymph node metastasis (LN1)-derived EVs in OSCC dissemination and metastasis. MS-based proteomics, lipidomics, and miRNA sequencing were run in processing triplicates for EVs from SCC-9 and LN1 cell lines to capture random biological variation. Proteomics analysis performed previously in our group showed that cell experiments in processing triplicate offer a good reliability of measurements and statistical power (53). To avoid random noise associated with protocols or equipment, proteomics data were also acquired in technical triplicates for each processing replicate and averaged. Considering that metabolite distributions are subjected to an enormous temporal and spatial variability (54), metabolomics experiments were carried out in processing quintuplicate, as described before in the literature (55). The log2 intensities from the multi-omics analysis are normally distributed and a two-sided Student's *t* test was used in Perseus v. 1.3.0.4 software (34) to determine proteins, miRNAs, lipids, and metabolites differentially abundant between LN1 and SCC-9 EVs (*p*-value ≤ 0.05). To avoid reducing the number of differentially abundant molecules and failing to perform process enrichment and the multi-omics analysis, we considered a nonadjusted *p*-value obtained from Student's *t* test and filtered out the proteins, miRNA, lipids, or metabolites using other strategies, like (i) filtering the datasets by a minimum of two processing replicates in at least one condition; (ii) performing the integrative analysis of the multi-omics, and (iii) for the proteomes, carrying out a comprehensive search of prognostic markers using public databases. PCA, hierarchical cluster heat map, subcellular location analysis, and biological processes/pathways enrichment were used to characterize the group of molecules associated with the metastatic behavior (33, 34, 36, 37, 38). An integrative multi-omics approach revealed central ‘hub proteins’, whose transcript levels were retrieved from public databases and associated with clinical information from patients with cancer. The public gene expression data were tested for normality and homogeneity of variance using the Shapiro–Wilk test to drive decisions of parametric (Student's *t* test and ANOVA) or nonparametric (nonadjusted Wilcoxon and Kruskal–Wallis) tests for group comparison with clinical categories (46). An AUC higher than 70% was set as the decision threshold to discriminate patients in the ROC curve. For survival analysis, *p*-values were obtained by log-rank tests for Kaplan–Meier curves in the HNCDB (47), PROGgeneV2 (48), and the Human Protein Atlas (52) databases using gene expression information from patients with cancer. A *p*-value ≤ 0.05 was used to determine significance in all statistical analyses.

## Results

### SCC-9 and LN1 EVs Show a Similar Size, Morphology, and Expression of Markers

**Fig. 1:**
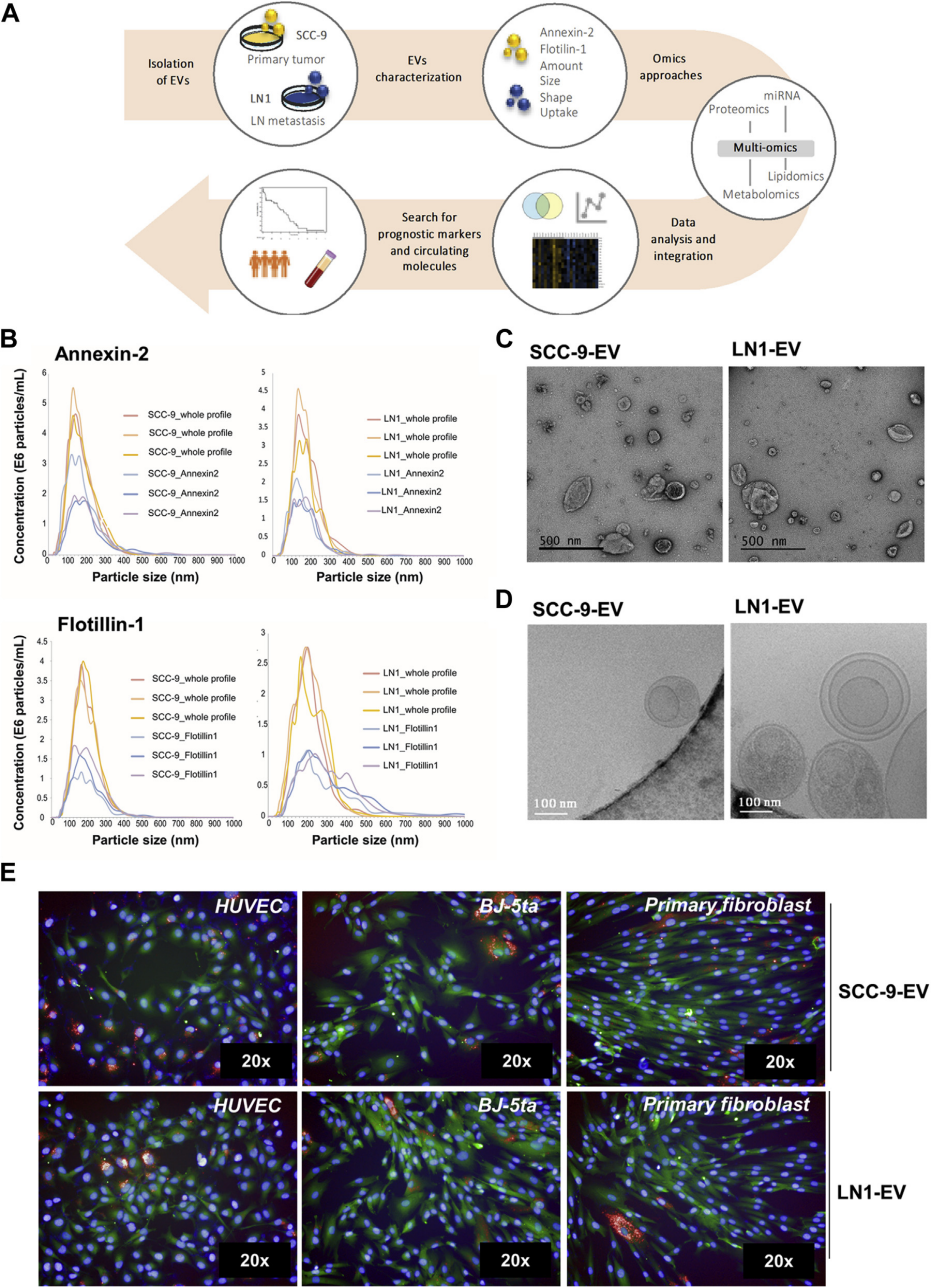
**Experimental design and characterization of EVs isolated from primary tumor (SCC-9 EVs) and metastatic (LN1 EVs) OSCC cell lines**. EVs were isolated from SCC-9 and LN1 cell lines, characterized, and submitted to a multi-omics approach, as shown in *panel A*. Annexin-2 and Flotillin-1 were identified in EVs by NTA-based antibody for both cell lines (*B*). Morphological characterization was performed by transmission electron microscopy and cryo-EM (*C* and *D*, respectively). HUVEC (human endothelial), BJ-5ta (human fibroblast), and primary fibroblast cells lines were used as recipient cells in internalization assays for SCC-9 and LN1 EVs to demonstrate the ability of EV internalization by different cell lines (*E*). The EVs are shown in *red* (DiD, Em. 660 nm), nuclei are in *blue* (DAPI, Em. 488 nm), and cytoplasm are labeled in *green* (CMFDA, Em. 517 nm). cryo-EM, cryo-electron microscopy; DAPI, 4′, 6-diamidino-2-phenylindole; DiD, 1,1′-dioctadecyl-3,3,3′,3′-tetramethylindodicarbocyanine, 4-chlorobenzenesulfonate salt; EVs, extracellular vesicles; NTA, nanoparticle tracking analysis; OSCC, oral squamous cell carcinoma.

In the present work, we investigated the differences between the molecular repertoire of EVs released by primary tumor (SCC-9) and metastatic cells (LN1), both derived from OSCC of the tongue, using a multi-omics approach followed by integrative analysis (Fig. 1*A*). SCC-9 EVs and LN1 EVs were isolated by ultracentrifugation, and the molecular and morphological characterization were performed by NTA and high-resolution microscopy, respectively. SCC-9 EVs showed a mean size of 172.6 ± 83.9 nm, whereas LN1 EVs had a mean size of 148.1 ± 59.4 nm. The protein markers Annexin-2 and Flotillin-1, usually identified in exosomes and microvesicles, were detected in the isolated EVs (Fig. 1*B*). We observed that Annexin-2 was detected in approximately 50% of the EVs isolated from SCC-9 and LN1 cells, whereas Flotillin-1 was detected in around 56% of the EVs isolated from SCC-9 cells and 68% of the EVs isolated from LN1 cells. It is important to note that this method only detects the proteins that have an antigen sequence accessible/exposed in the external surface of the EVs, and it is likely that a higher percentage of EVs may carry these molecules. Besides, the EVs showed a specific shape and size under transmission electron microscopy and cryo-EM (Fig. 1, *C* and *D*, respectively). The qualitative cryo-EM analysis revealed EVs with smooth surfaces and no internal density, as well as vesicles with electron-dense internal granules. Moreover, we observed EVs with a multilayered pattern and some vesicles with spikes extending from the membrane, most likely proteins.

### The Isolated EVs Are Active and Can Be Internalized by Recipient Cells

Considering that EVs must be taken up by recipient cells to have a role in intercellular communication, we incubated EVs isolated from SCC-9 and LN1 cells with endothelial (HUVEC) and fibroblast (BJ-5ta and primary culture) recipient cells for 48 h. Microscopy analysis revealed that EVs from both cell lines could be internalized by recipient cells and are mostly located in the perinuclear region (Fig. 1*E*). Between 11.0% and 15.0% of HUVEC and BJ-5ta cells showed internalized EVs derived from SCC-9 and LN1 cells, respectively (supplemental Fig. S1). However, the number of primary fibroblasts containing internalized EVs was highly discrepant for SCC-9 EVs and LN1 EVs: while 17.0% of primary fibroblasts could uptake SCC-9-derived EVs (supplemental Fig. S1*A*), only 2.3% of the cells had LN1 EV internalized (supplemental Fig. S1*B*). By this analysis, we confirmed that the isolated EVs released by primary tumor and metastatic oral cancer cells can be selective regarding internalization, showing more or less uptake by recipient cell lines.

### EVs From OSCC Cells Carry Proteins Associated with the Metastatic Behavior

**Fig. 2:**
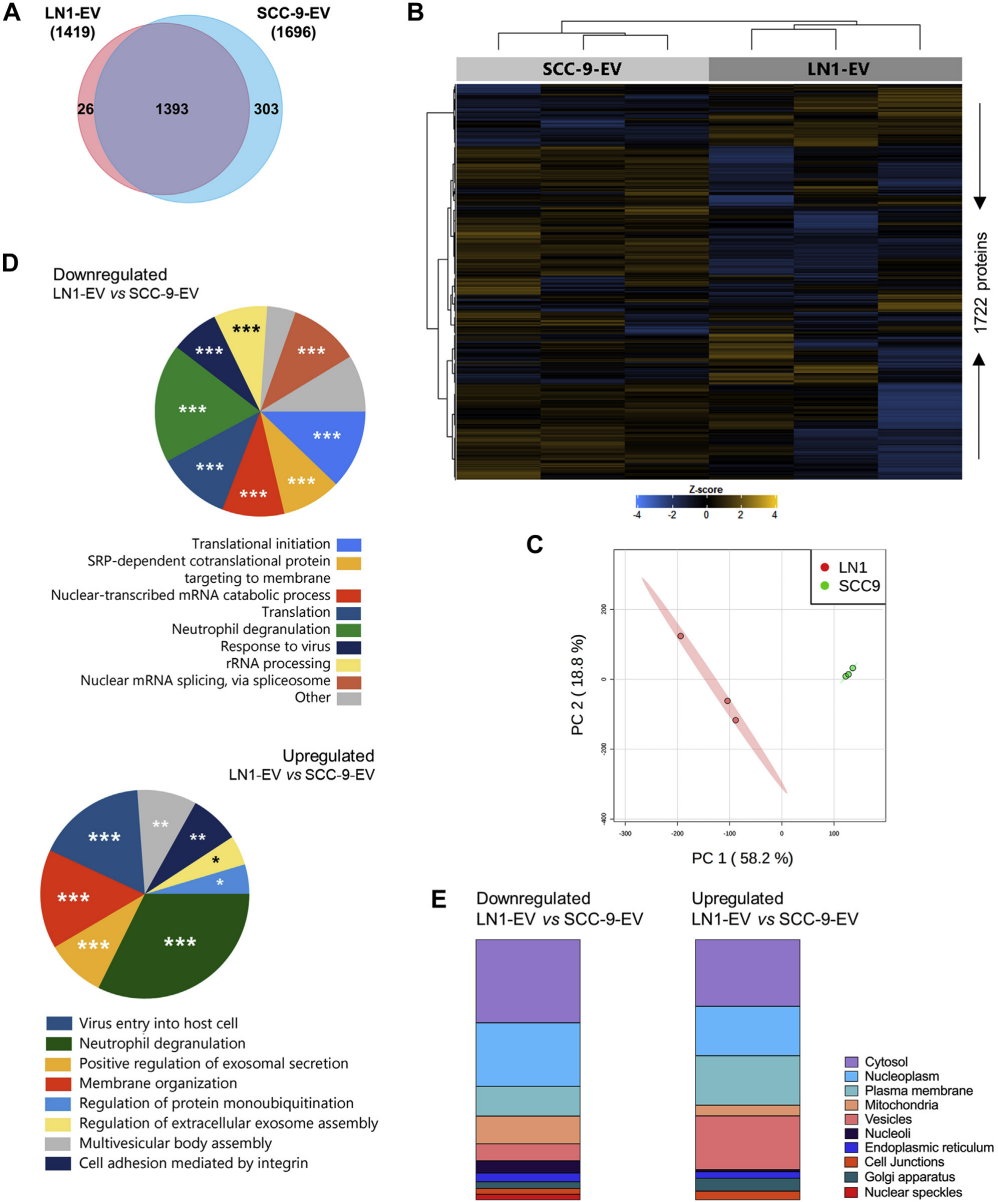
**Proteome characterization of SCC-9-and LN1-derived EVs.** The overlap between proteins identified for EVs isolated from primary tumor and lymph node metastatic cells is shown in the Venn diagram (*A*). Unsupervised hierarchical cluster heat map (*B*) and PCA plot (*C*) show grouping of replicates according to the proteomic profile. The cluster was generated using the Euclidean distance and complete linkage method (n = 1722 proteins). The top ten GO biological processes and subcellular location enriched for the differentially abundant proteins between the two groups are shown in *panels D* and *E*, respectively. ∗*p*-value ≤ 0.05; ∗∗*p*-value ≤ 0.01; ∗∗∗*p*-value ≤ 0.001 (*p*-values were determined using the Bonferroni method). EVs, extracellular vesicles; GO, Gene Ontology; PCA, principal component analysis.

Aiming to identify the protein composition of OSCC-derived EVs associated with lymph node metastasis, we determined the proteomic profile of EVs isolated from SCC-9 and LN1 cell lines using an LTQ Orbitrap Velos mass spectrometer (Thermo Fisher Scientific). Detailed information for all peptides and proteins identified is presented in supplemental Tables S1 and S2. The exclusion of entries assigned as ‘Reverse’ and ‘Only identified by site’ and filtering for two valid values in at least one group resulted in 1696 proteins identified for SCC-9 EVs and 1419 proteins for LN1 EVs (Fig. 2*A*), which could classify EVs into two distinct groups, as shown in the heat map and PCA plot (Fig. 2, *B* and *C*; supplemental Table S3).

Six hundred 70 proteins were differentially abundant between LN1 EVs and SCC-9 EVs, with 140 upregulated (21%) and 530 downregulated (79%) in LN1 EVs compared with SCC-9 EVs (Student's *t* test; *p*-value ≤ 0.05) (supplemental Table S4). By using FunRich tool and GO annotation, in general, we observed that proteins downregulated in EVs from LN1 were involved in translation and transcription processes, including translational initiation (*p*-value = 7.960E-13), rRNA processing (*p*-value = 0.0001), and nuclear RNA splicing (*p*-value = 0.0002), whereas upregulated proteins in LN1 EVs may play a role in exosome assembly and secretion through positive regulation of exosomal secretion (*p*-value = 8.234E-06), multivesicular body assembly (*p*-value = 0.0009), and regulation of extracellular exosome assembly (*p*-value = 0.018), as well as in cell adhesion mediated by integrin (*p*-value = 0.0045) (Fig. 2*D*). Interestingly, both downregulated and upregulated datasets have a significant number of inflammatory proteins, which is represented by the enrichment of neutrophil degranulation process (8.3% of proteins upregulated in LN1 EVs, *p*-value = 1.286E-06 and 16% of downregulated proteins, *p*-value = 6.381E-07).

We also evaluated the subcellular location of differentially abundant proteins using the Human Protein Atlas database and found that downregulated proteins are mainly related to proteins located in the cytosol (n = 131 proteins), nucleoplasm (n = 61), and plasma membrane (n = 57), whereas upregulated proteins were identified more frequently in the cytosol (n = 31), followed by vesicles (n = 25), nucleoplasm (n = 23), and plasma membrane (n = 23) (Fig. 2*E*).

### LN1 and SCC-9 EVs Mostly Reflect the Content of Their Precursor Secreting Cells

To correlate the composition of EVs with their progenitor cells, we performed proteomics analysis of SCC-9 and LN1 whole cells. After excluding ‘Reverse’ sequences and ‘Only identified by site’ entries and filtering for two valid values in at least one group, 2705 proteins were identified for SCC-9 and LN1 parental cells, 818 of them differentially abundant between the two groups (Student's *t* test; *p*-value ≤ 0.05) (supplemental Tables S5–S8). About 83.7% and 83.6% of all proteins carried by LN1 and SCC-9 EVs were shared with LN1 and SCC-9 whole cells, respectively, indicating that LN1 and SCC-9 EVs mostly reflect the composition of the origin cells (supplemental Fig. S2*A*; supplemental Tables S9–S12). The enrichment analysis using FunRich software against the GO database showed that both LN1 and SCC-9 whole cells and EV proteomes are involved in the same biological processes, indeed confirming the similarity between progenitor cells and EVs (supplemental Fig. S2*B*). LN1 and SCC-9 cells and EVs significantly (*p*-value ≤ 0.05) carry proteins associated with inflammation (neutrophil degranulation; 7.8% and 11.8% of proteins identified in LN1 cells and EVs, 7.8% and 10.7% of proteins identified in SCC-9 cells and EVs, respectively), viral reproduction (7.4% and 8.0% of proteins identified in LN1 cells and EVs, 7.4% and 7.6% of proteins identified in SCC-9 cells and EVs, respectively), signal transduction (4.4% and 5.7% of proteins identified in LN1 cells and EVs, 4.4% and 5.4% of proteins identified in SCC-9 cells and EVs, respectively), and translational initiation (4.8% and 6.9% of proteins identified in LN1 cells and EVs, 4.8% and 6.1% of proteins identified in SCC-9 cells and EVs, respectively). Only proteomes from LN1 cells and EVs are enriched for nuclear-transcribed mRNA catabolic process (3.9% and 5.0% of proteins identified in LN1 cells and EVs, respectively), while SCC-9 cells and EV content is uniquely involved in post-translational protein modification (4.3% and 5.5% of proteins identified in LN1 cells and EVs, respectively) (*p*-value ≤ 0.05).

### LN1-Derived EVs Display an miRNA, Metabolic, and Lipid Profile Associated with Tumor Dissemination

To better explore the composition of OSCC-derived EVs that is associated with lymph node metastasis, we evaluated the miRNA, metabolomic, and lipidomic profiles of SCC-9- and LN1-derived EVs.

**Fig. 3:**
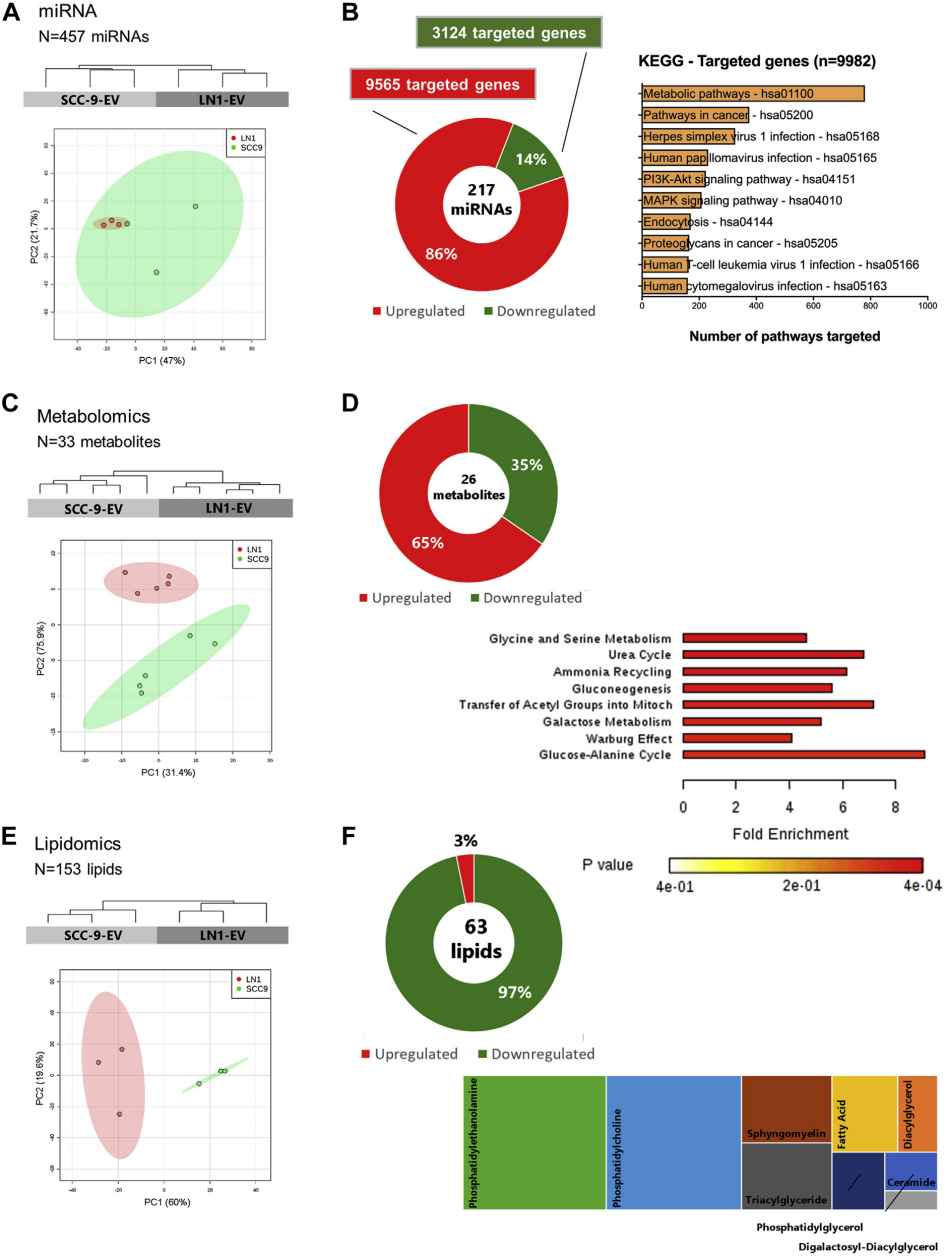
**miRNA, metabolomics, and lipidomics analysis of EVs isolated from SCC-9 and LN1 cell lines**. Sample grouping according to miRNA, metabolite, and lipid profiles are shown in the dendrograms (*upper panel*) and PCA plots (*lower panel*) for SCC-9 EV and LN1 EV replicates (*A*, *C*, and *E*, respectively). The dendrograms were generated using Euclidean distance and complete linkage method (n = 457 miRNAs, 33 metabolites, and 153 lipids). Differentially expressed miRNAs can target specific genes that are significantly mapped to KEGG pathways (*B*; *p*-value ≤ 0.05). Differentially expressed metabolites are associated with specific biological processes (*D*; *p*-value ≤ 0.05). The class composition of differentially abundant lipids between SCC-9 and LN1-derived EVs is shown in *panel F*. EVs, extracellular vesicles; KEGG, Kyoto Encyclopedia of Genes and Genomes.

The miRNA analysis resulted in a list of 457 miRNAs identified for EVs isolated from SCC-9 and LN1 (supplemental Table S13). In agreement with proteomics datasets, the miRNA profile could also classify primary tumor and metastasis EVs in two groups using PCA and hierarchical clustering analysis (Fig. 3*A*). Two hundred 17 miRNAs were differentially expressed between LN1 EVs and SCC-9 EVs (187 upregulated and 30 downregulated) (Fig. 3*B*; supplemental Table S14). The upregulated miRNAs can modulate 9565 targeted genes, whereas downregulated miRNAs may target 3124 genes (supplemental Table S15). By using KEGG database, we showed that the targeted genes are mapped mainly to metabolic pathways (n = 780 pathways), followed by pathways in cancer (n = 375 pathways) (Fig. 3*B*). It is important to highlight that miRNA may modify genes involved in two important cancer-related signaling pathways: PI3K-Akt (n = 223 pathways) and MAPK (n = 208 pathways).

Thirty-one metabolites were identified for SCC9-EVs and 33 metabolites for EVs isolated from LN1 cell line (supplemental Table S16). The metabolic profile could also separate primary tumor and metastatic EVs in two distinct groups (Fig. 3*C*). Twenty-six metabolites were differentially abundant between LN1- and SCC-9-derived EVs (17 upregulated and nine downregulated) (Fig. 3*D*; supplemental Table S17). The enrichment analysis showed that the differentially abundant compounds may be involved in eight biological processes (*p*-value ≤ 0.05), mainly glycine/serine metabolism and urea cycle (*p*-value = 0.0271) (Fig. 3*D*). It is interesting to note an overrepresentation of metabolites involved in the Warburg effect, a phenomenon that occurs in most cancer cells (*p*-value = 0.0338).

Lipid repertoire of SCC-9 EVs (n = 151) and LN1 EVs (n = 123) could also separate EVs into distinct groups (Fig. 3*E*; supplemental Table S18). Sixty-three lipids were differentially abundant between LN1- and SCC-9-derived EVs (61 downregulated and two upregulated), most of them belonging to phosphatidylethanolamine (PE, n = 19 compounds) and phosphatidylcholine (PC, n = 18 compounds) classes (Fig. 3*F*; supplemental Table S19). Thus, we found a set of PE and PC compounds downregulated in OSCC-EVs isolated from metastatic cells when compared with primary tumor-derived EVs.

### An Integrative Analysis Reveals Associated Molecules Across Multiple Omics Datasets in OSCC-Derived EVs

**Fig. 4:**
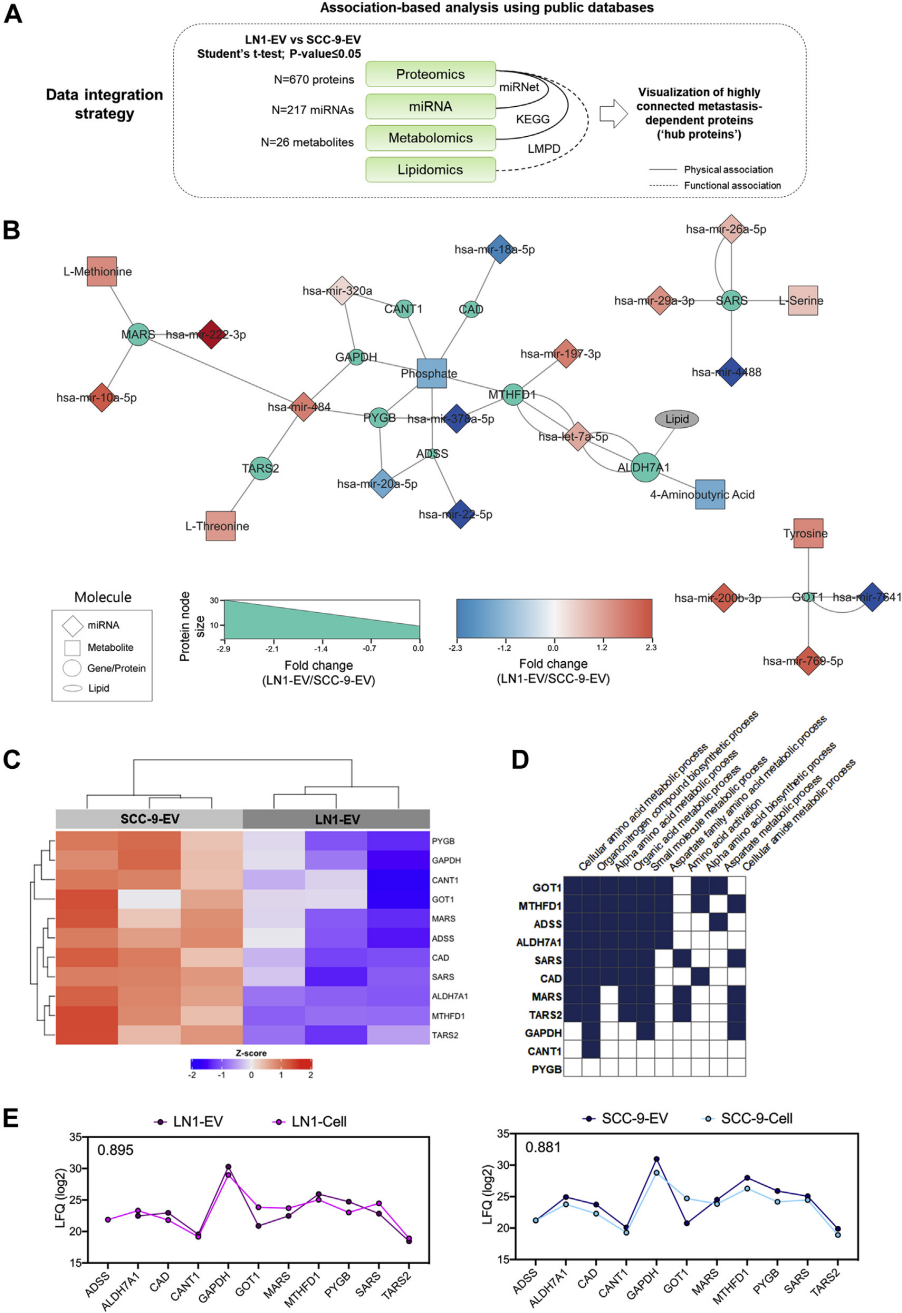
**Multi-omics integrative analysis of proteomics, miRNA, metabolomics, and lipidomics content from SCC-9 and LN1-derived EVs**. The integration of differentially abundant molecules from EVs (Student's *t* test; *p*-value ≤ 0.05; LN1 EVs *versus* SCC-9 EVs) based on associations (*A*) showed as a set of 11 ‘hub proteins’ that interact with specific miRNAs or metabolites, as well as the presence of the lipid-associated protein ALDH7A1 (*B*). All ‘hub proteins’ are downregulated in LN1 EVs when compared with SCC-9 EVs (*C*) and may modulate specific biological processes associated with metabolism (*D*). The heat map was generated using the Euclidean distance and complete linkage method (n = 11 proteins). The correlation between protein abundance in cells and EVs was calculated for the 11 selected proteins considering SCC-9 and LN1 protein intensities (*E*). The Pearson coefficient is shown in the upper left corner of the graphs. EVs, extracellular vesicles.

We next used a multi-omics integrative approach to determine EV molecules involved in lymph node metastasis that are highly connected with other molecules among our omics datasets. For that, we used as input the significant proteins, miRNA, and metabolites from OSCC-EVs associated with lymph node metastasis (LN1 EVs compared with SCC-9 EVs; Student's *t* test, *p*-value ≤ 0.05; n = 670 proteins, 217 miRNAs, 26 metabolites) and considered physical associations between the datasets. In addition, differential proteins functionally related to lipid processes and pathways were included in the workflow (Fig. 4*A*).

Using the association-based integrative approach, we revealed 11 differentially abundant proteins between LN1 EVs and SCC-9 EVs (ADSS, ALDH7A1, CAD, CANT1, GAPDH, GOT1, MARS, MTHFD1, PYGB, SARS, and TARS2) that interact with 16 miRNA (hsa-let-7a-5p, hsa-miR-10a-5p, hsa-miR-18a-5p, hsa-miR-197-3p, hsa-miR-22-5p, hsa-miR-222-3p, hsa-miR-320a, hsa-miR-378a-5p, hsa-miR-4488, hsa-miR-484, hsa-miR20a-5p, hsa-miR-29a-3p, hsa-miR-26a-5p, hsa-miR-200b-3p, hsa-miR-769-5p, and hsa-miR-7641) and six metabolites (4-aminobutyric acid, L-methionine, L-serine, L-threonine, phosphate, and tyrosine) also dysregulated in our datasets (Fig. 4*B*, supplemental Table S20). In addition, ALDH7A1 is listed as a lipid-associated protein according to LMPD and may have a relationship with the lipidomics content of EVs. The 11 proteins were considered highly connected in our study and named ‘hub proteins’ in subsequent analysis. The ‘hub proteins’ were significantly downregulated in the metastatic site (LN1-derived EVs) when compared with the primary site (SCC-9-derived EVs) and are enriched for metabolic processes (*p*-value ≤ 0.05) (Fig. 4, *C* and *D*, respectively).

In summary, our multi-omics integrative strategy revealed a signature of 11 central ‘hub proteins’, which are modulated in the metastatic site EVs (LN1 EVs) when compared with primary site EVs (SCC-9 EVs) derived from OSCC. These proteins were used as the input to further prospect molecules from our dataset that may be used as prognostic markers.

### Targeted Proteins Derived From Multi-Omics Analysis Have a Role in Prognosis

We observed that the 11 central molecules show high Pearson correlation coefficient when comparing the mean intensities of each protein between EVs and progenitor cells SCC-9 and LN1 (r = 0.895 for LN1 cells and EVs; r = 0.881 for SCC-9 cells and EVs) (Fig. 4*E*). Considering that these results indicate that EVs may reflect the content of the origin cell, we next translated the EV findings to predict patient outcome using tumoral tissue information from public repositories. For that, the gene expression profile of input transcripts from the 11 ‘hub proteins’ was compared with clinical and pathological information of patients with OSCC, HNSCC, and other cancers using information retrieved from the public databases TCGA, GSE41613, E-MTAB-1328, and GSE65858.

**Fig. 5:**
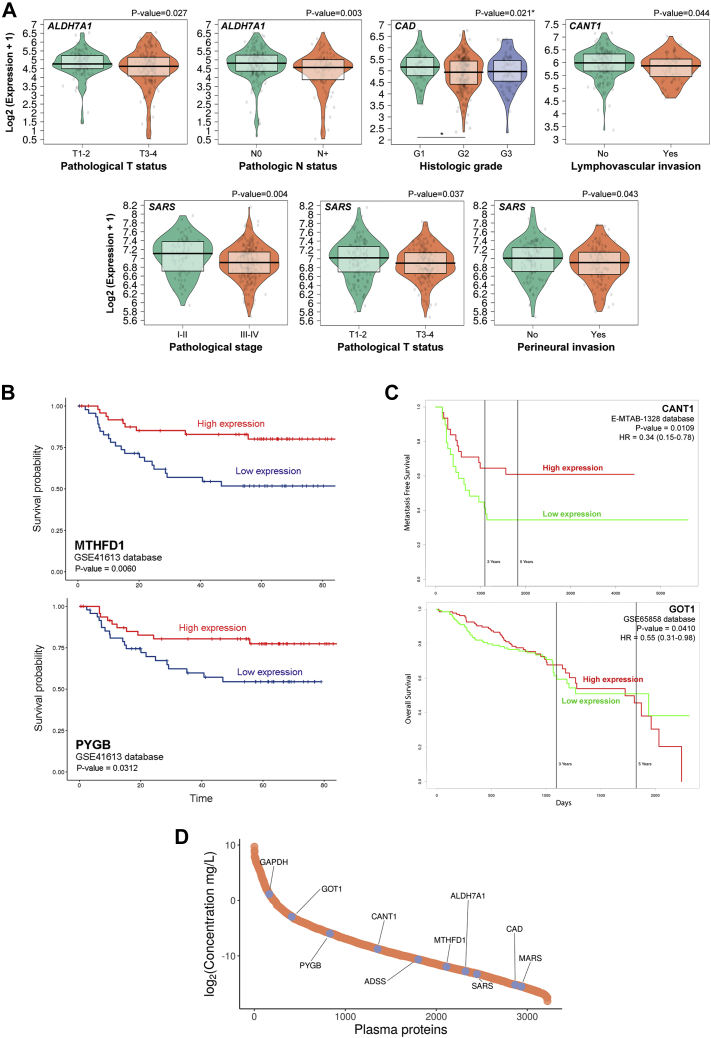
**Characterization of prognostic markers in HNSCC using public databases**. TCGA, GSE41613, GSE65858, and MTAB-1328 repositories were used to determine proteins from the multi-omics integrative analysis associated with clinical and pathological features in patients with HNSCC. Using TCGA database, the gene expression pattern of a group of hub proteins was significantly associated with clinical features (*A*; *ALDH7A1*, *CAD*, *CANT1*, *SARS*, *p*-value ≤ 0.05). *MTHFD1* and *PYGB* downregulation was linked to poor overall survival in GSE41613 database using HNCDB tool (*B*), whereas *CANT1* and *GOT1* low transcript levels were associated with poor overall survival and metastasis-free survival in GSE-65858 and E-MTAB-1328 databases, respectively (*C*) (*p*-value ≤ 0.05). The concentration of plasma proteins from healthy humans was retrieved for the 11 ‘hub proteins’ from MS experiments available in The Human Plasma Proteome and represented in a dynamic range plot (*D*). The proteins of interest are highlighted in the graph. HNCDB, Head and Neck Cancer Database; HNSCC, head and neck squamous cell carcinoma; N0, patient negative for lymph node metastasis considering pathological staging; N+, patient positive for lymph node metastasis considering pathological staging; TCGA, The Cancer Genome Atlas.

Considering data from TCGA repository for OSCC primary tumors, lower gene expression of *ALDH7A1* (*p*-value = 0.027) and *SARS* (*p*-value = 0.037) were associated with advanced T status, while the downregulation of transcripts *ALDH7A1* (*p*-value = 0.003), *CAD* (*p*-value = 0.021), *CANT1* (*p*-value = 0.044), and *SARS* (*p*-value = 0.004 and 0.043) correlated with the presence of lymph node metastasis, advanced histologic grade, presence of lymphovascular invasion, advanced stage and positivity for perineural invasion, respectively (Fig. 5*A*; supplemental Table S20). The distribution of transcript levels for each clinical group was used to evaluate normality (Shapiro–Wilk test) and guided the statistical decisions to investigate the association between gene expression of ‘hub proteins’ and prognostic features (supplemental Fig. S3; supplemental Table S20).

The downexpression of ‘hub proteins’ in primary tumor tissue was also associated with survival in patients with HNSCC using GSE41613, E-MTAB-1328, and GSE65858 databases. Lower transcript levels of *MTHFD1* and *PYGB* genes determined poor overall survival in patients with HNSCC (*p*-value = 0.006 and *p*-value = 0.0312, respectively) using the HNCDB (Fig. 5*B*; supplemental Table S20), while lower gene expression of *CANT1* and *GOT1* was associated with reduced metastasis-free survival and overall survival, respectively, according to PROGgeneV2 tool (*p*-value = 0.0109 and *p*-value = 0.0410) (Fig. 5*C*; supplemental Table S20).

To evaluate whether the 11 ‘hub proteins’ could define prognosis in other tumor types, we compared transcript levels between primary tumor and metastasis for multiple cancer types using TCGA datasets. Downregulation of *GOT1* (*p*-value = 0.019) was found in the metastasis site in THCA (supplemental Fig. S4, *A* and *B*; supplemental Table S20). *GOT1* could also accurately classify samples according to the tumor site in THCA (ROC curve; AUC = 73.6%). Besides that, low transcript levels retrieved from TCGA primary tumors for three of the 11 ‘hub proteins’ (*ALDH7A1*, *GOT1*, *MTHFD1*) were associated with poor overall survival in renal and cervical cancer (*p*-value ≤ 0.05; Human Protein Atlas platform) (supplemental Fig. S4*C*; supplemental Table S20).

These data show that transcripts from seven proteins prioritized in our multi-omics approach, ALDH7A1, CAD, CANT1, GOT1, MTHFD1, PYGB and SARS, are associated with prognosis in HNSCC or other tumor types and may be further evaluated as clinical markers in the disease.

### Proteins Prioritized in Integrative Analysis May Circulate Throughout the Body

Considering the 11 ‘hub proteins’ that were identified in EVs and correlated with patient outcome, we next analyzed if this set of proteins could circulate throughout the body and be used as markers in liquid biopsies. For that, we evaluated the presence and concentration of our selected proteins in human normal plasma using MS information available in the Human Plasma Proteome database. Ten from the 11 ‘hub proteins’ were identified and quantified in human plasma with concentrations ranging from 21 ng/L (MARS) to 2.2 mg/L (GAPDH). Besides GAPDH, the most abundant proteins from our dataset were GOT1 (130 μg/L), PYGB (16 μg/L), and CANT1 (2.3 μg/L) (Fig. 5*D*; supplemental Table S20).

Altogether, besides elucidating tumor EV physiology and behavior, our multi-omics approach indicated ‘hub proteins’ in EVs that are associated with prognosis and detected in whole blood.

## Discussion

Shed EVs are key elements in intercellular communication and can cause a significant impact on cancer development and progression through the transfer of molecules to recipient cells (56). Emerging evidences indicate that tumor-derived EVs can play a role in the metastatic process (9, 10, 13), and detection of changes within vesicle cargoes is of special interest for cancer diagnosis, prognosis, and monitoring (57). Herein, we elucidated for the first time the molecular aspects associated with the metastatic phenotype in OSCC through assessing the proteomic, miRNA, metabolomic, and lipidomic profiles of EVs isolated from human primary tumor (SCC-9 cells) and matched lymph node metastasis (LN1 cells) that was established using an orthotopic mouse model (20). As far as we know, there are not any additional paired human primary tumor and lymph node metastasis cell lines established for oral cancer or other tumors. We developed a multi-omics integrative analysis and determined a set of ‘hub proteins’ from EVs that interact with dysregulated miRNAs and metabolites from our datasets and correlated with aggressiveness and prognosis in HNSCC.

By using a reductionist approach and comparing the protein profile from the two cell lines, we found that metastatic site–derived EVs (LN1 EVs) have an overrepresentation of molecules involved in cell adhesion mediated by integrin. Interestingly, a previous study using lung, liver, and brain-tropic tumor cells showed that integrins inserted in the membrane of exosomes directed the colonization of specific organs by fusing with target cells in a tissue-specific manner (13). The authors revealed that integrin expression profiles correlated with tissue organotropism, specifically ITGA6, ITGB4, ITGB1, ITGB5, and ITGαv, and targeting specific integrins decreased exosome uptake and distant metastasis. In the case of our study, we identified the upregulation of a different set of integrins in LN1 EVs originated from the site of metastasis (ITGB1, ICAM1, ITGB6, ITGAV, and ITGA5), adding the knowledge that besides the role on organotropism to a specific tissue, the established tissue also releases EVs with this signature, increasing the possibility of targeting these molecules as a therapy for both regional and distant metastasis.

On the other hand, it is interesting that 79% of the proteins are in lower abundance in LN1 EVs when compared with SCC-9 EVs, that is, upregulated in primary tumor OSCC EVs. These molecules are essentially associated with transcription and translation, which are key cellular processes. It is true that primary tumor cells need to modify recipient cells in an extensive way, for example, to prepare future sites for lymph node metastasis. In fact, the literature shows that EVs shed by tumor cells can precondition the microenvironment of organs where metastases will develop to make them receptive for disseminating tumor cells, the premetastatic niche concept (58). For instance, EVs from melanoma and pancreatic cancer cells have been shown to orchestrate a variety of lymph node premetastatic changes *in vivo*, including extracellular matrix deposition and vascular proliferation (59, 60). In our dataset, SCC-9 EVs are overrepresented in RNA-splicing proteins (*p*-value = 0.0002) and may highly modulate recipient cells through this process. Several studies show that the splicing machinery and transcripts produced are significantly changed in cancer (61), but the contribution of EVs in such alterations is poorly described. Also, it was proposed that the study of tumor EV surface landscape is essential for the detection of cancer-specific exons derived from alternative splicing events that may be used in EV-targeted therapies (62).

Remarkably, we found for the first time a miRNA signature correlated with OSCC dissemination in EVs that could target genes involved in PI3K-Akt and MAPK signaling pathways in recipient cells. Both pathways regulate key cellular processes, including cell proliferation, survival, and growth, and their aberrant activation is frequently detected in many types of cancer (63, 64). Actually, multiple drugs targeting PI3K-Akt and MAPK signaling pathway have been developed (64, 65). Most of the studies indicate the regulation of these pathways through the secretion of EVs from primary cells. In nasopharyngeal carcinoma cell-derived EVs, the secretion of PIK3CA was evidenced and phosphorylates PIP2 as the first step of PI3K-Akt signaling pathway (66). Besides, the transport of the epidermal growth factor receptor *via* EVs from bladder, colorectal, and brain tumor cells (67, 68, 69) can also activate the PI3K-Akt pathway. It was shown that gastric cancer–derived EVs can increase pAkt and proliferation of recipient cells (70), and transferrin receptor 2 is released by hepatoblastoma and erythroleukemia-derived exosomes and activates signal transduction through the MAPK pathway (71). Our data indicate that miRNAs carried by OSCC EVs, in which levels correlate with metastasis potential, may also target these relevant signaling pathways.

Alteration of metabolism is one of the hallmarks for cancer progression (72), and metabolites are a surrogate of the physiological/phenotypic state of the cell, making them an ideal way to track changes and mine for potential biomarkers. In this work, metabolomics analysis revealed that metabolites associated with nodal metastasis in OSCC EVs are overrepresented in aminoacyl-tRNA biosynthesis pathway and the Warburg effect biological process. Aminoacyl-tRNAs are substrates for translation and are pivotal in determining how the genetic code is interpreted as amino acids (73), so that OSCC EVs from primary and metastatic sites may play a differential role in transcription through the delivery of metabolite cargo to recipient cells. Although the Warburg effect has been well documented for cancer cells, the role of EVs in this process is poorly known. However, it was shown that exosomes derived from stroma tissue, the cancer-associated fibroblasts, could promote glycolysis and block oxidative metabolism in prostate cancer cells, interfering in the Warburg effect (74). Therefore, interestingly, our data showed that a distinct dysregulation in metabolites resulted from the Warburg effect in EVs isolated from metastatic cells when compared with primary OSCC cells, which may result in a distinct role of both cell lines in affecting glycolysis in recipient cells and reflect the cell type phenotype.

To complete the omics analysis, considering the role of lipids in energy storage, signaling and as structural components, we performed lipidomic analysis. This is a relatively recent research field that has been driven by rapid advances in several analytical technologies, in particular MS, computational methods, and it is recognized with a role in many diseases (75). In this study, most of the lipids differentially abundant between lymph node metastasis and primary tumor OSCC EVs belonged to PE and PC classes. PC and PE are phospholipids, which serve as building blocks for cellular membranes (lipid bilayer) and are involved in a diverse array of functions such as cell signaling and execution of both cellular proliferation and death programs (76, 77, 78). Notably, in this work, we found a relation between lower abundance of PE and PC and lymph node metastasis–derived EVs.

Collectively, through a multi-omics integrative approach for proteomic, miRNA, metabolomic, and lipidomic data based on functional or physical associations described in miRNet (35), KEGG (36), and LMPD (39) databases, we identified 11 ‘hub proteins’ (ADSS, ALDH7A1, CAD, CANT1, GAPDH, GOT1, MARS, MTHFD1, PYGB, SARS, and TARS2) associated with metabolism and nodal metastasis in our proteomics dataset (LN1 EVs *versus* SCC-9 EVs). These proteins were downregulated in metastatic EVs (LN1 EVs) when compared with primary site EVs (SCC-9 EVs), showing that low protein abundances were associated with the most aggressive phenotype, as reported previously (20). The authors showed that the metastatic cell line LN1 seems to be more invasive *in vitro* than the primary tumor parental cell line SCC-9 when using a myoma organotypic invasion assay (20). Remarkably, low transcript levels of seven from 11 ‘hub proteins’ (*ALDH7A1*, *CAD*, *CANT1*, *GOT1*, *MTHFD1*, *PYGB*, and *SARS*) were associated with a more aggressive clinical outcome, that is, poor patient prognosis, using data from patients with HNSCC from the public databases TCGA, GSE41613, E-MTAB-1328, and GSE65858. The downregulation of the transcripts *ALDH7A1*, *GOT*, and *MTHFD1* also correlated with poor prognosis in patients with other tumors.

The association of ‘hub proteins’ from the multi-omics integrative analysis with prognosis features was based on gene expression information from cancer tissues available in public databases, once protein abundance information and clinical features for patients with cancer are not publicity available in the literature. A Pearson correlation analysis showed high correlation coefficient (r > 0.8) for the 11 ‘hub protein’ intensities between EVs and cells from SCC-9 and LN1, indicating that information obtained from cells or tissues in the public databases can be extrapolated for the 11 EV proteins. Besides that, protein levels may be largely determined by transcript concentrations under specific conditions and taking into account the recent improvement of techniques for RNA and protein identification (79). Thus, we decided to use gene expression public data to transcend the clinical significance of selected proteins and successfully prospect candidates as prognostic markers in cancer.

There are several advantages in using targets in EVs as prognosis markers instead of cells or free-molecule evaluation. These structures can preserve the constitution of the origin cells, as shown in this work, when comparing the proteome composition of SCC-9 and LN1 cells and EVs, with the advantage that the encapsulation of labile molecules such as RNA and proteins within lipid bilayers offers protection or decreases the rate of degradation. It is also interesting to note that ten molecules from our group of 11 ‘hub proteins’, including the seven proteins associated with prognosis, are candidates to circulate throughout the body according to MS data available in the Human Protein Atlas platform, which makes them strong candidates to be used as prognostic markers in a liquid biopsy context. Liquid biopsies hold a great promise for personalized medicine because of their ability to provide multiple noninvasive global snapshots of the primary and metastatic tumors.

In summary, by a reductionist approach, we characterized for the first time the molecular profile of EVs isolated from primary tumor and the paired metastatic site of oral cancer cell lines using proteomics, miRNA, metabolomics, and lipidomics, followed by an integrative strategy. By this analysis, we determined a set of molecules carried by EVs that are associated with metastasis and may modulate signaling pathways in recipient cells. The integrative multi-omics analysis leveraged the search for potential markers of lymph node metastasis and prospected a set of EV ‘hub proteins’ associated with aggressiveness in patients with cancer that potentially serve as prognostic markers in OSCC.

## Data availability

The mass spectrometry proteomics data have been deposited to the ProteomeXchange Consortium *via* the PRIDE (80) partner repository with the dataset identifier PXD019216. Annotated spectra can be consulted through MS-Viewer with search keys kry3zycx2j and i78ksoatlk (81). All FASTQ files of sRNA sequencing were deposited in the Sequence Read Archive (http://www.ncbi.nlm.nih.gov/sra/) and are available through the BioProject ID #PRJNA285452. Metabolomics data are available at the NIH Common Fund Data Repository and Coordinating Center (http://www.metabolomicsworkbench.org), under Metabolomics Workbench Study ID ST001755. Lipidomics data are intellectual property of metaSysX company, and the delivered tables comprising normalized and raw intensities of detected features per samples are attached in this article as supplemental data (Supplemental Tables S21 and S22, respectively).

## Supplemental data

This article contains supplemental data.

## Conflict of interest

The authors declare no competing interests.
