# Supplementary material for: A Reductionist Approach Using Primary and Metastatic Cell–Derived Extracellular Vesicles Reveals Hub Proteins Associated with Oral Cancer Prognosis
Source: Mol Cell Proteomics. 2021 Jun 27;20:100118. doi: 10.1016/j.mcpro.2021.100118 (PMC8350068; doi:10.1016/j.mcpro.2021.100118)
Supplement: Supplemental Figures S1–S4 [file mmc1.pdf]

Supplemental Figures

A reductionist approach using primary and metastatic cell-derived extracellular vesicles reveals hub proteins associated with oral cancer prognosis

Ariane Fidelis Busso-Lopes, Carolina Moretto Carnielli, Flavia Vischi Winck, Fábio Malta de Sá Patroni, Ana Karina de Oliveira, Daniela Campos Granato, Rute Alves Pereira e Costa, Romênia Ramos Domingues, Bianca Alves Pauletti, Diego Mauricio Riaño-Pachón, Juliana Aricetti, Camila Caldana, Edgard Graner, Ricardo Della Coletta, Kelly Dryden, Jay William Fox, Adriana Franco Paes Leme

Material included:

Supplemental Fig. S1. Uptake of EVs by recipient cells.

Supplemental Fig. S2. Comparison of the proteomic profile between SCC-9 and LN1 cells and EVs.

Supplemental Fig. S3. Evaluation of the normality assumption in transcript levels from hub proteins using TCGA database for OSCC.

Supplemental Fig. S4. Characterization of prognostic markers in multiple tumor types.

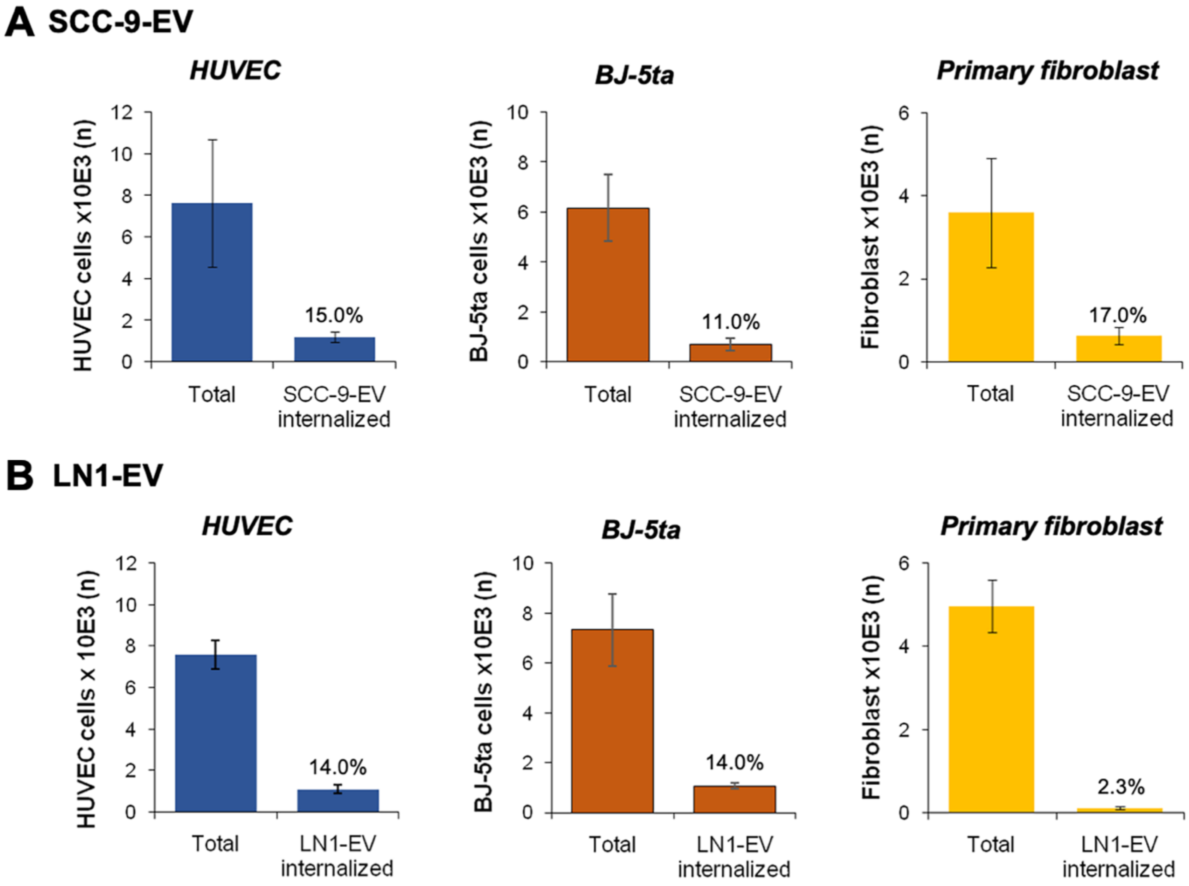

Supplemental Fig. S1. Uptake of EVs by recipient cells. The number of recipient cells with internalized SCC-9 and LN1-derived EVs is shown in (A) and (B), respectively. Three replicates were performed for every condition. Data are represented as mean ± SD.

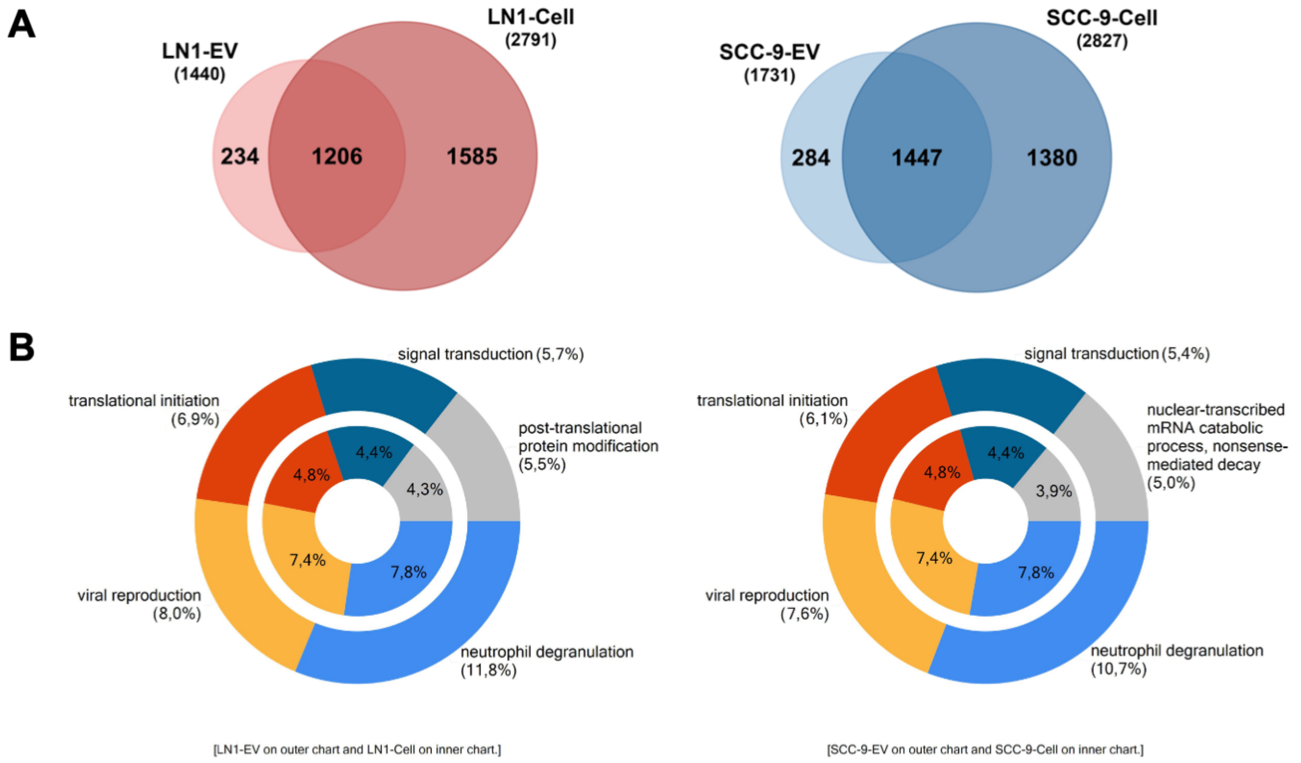

Supplemental Fig. S2. Comparison of the proteomic profile between SCC-9 and LN1 cells and EVs. The overlap between proteins identified for EVs and cells from primary tumor and lymph node metastasis cells is shown in the Venn diagram (A). The GO biological processes enriched for cells and EVs are represented in (B) (P-value≤0.05) and the percentages indicate the number of proteins in the dataset involved in a given biological process.

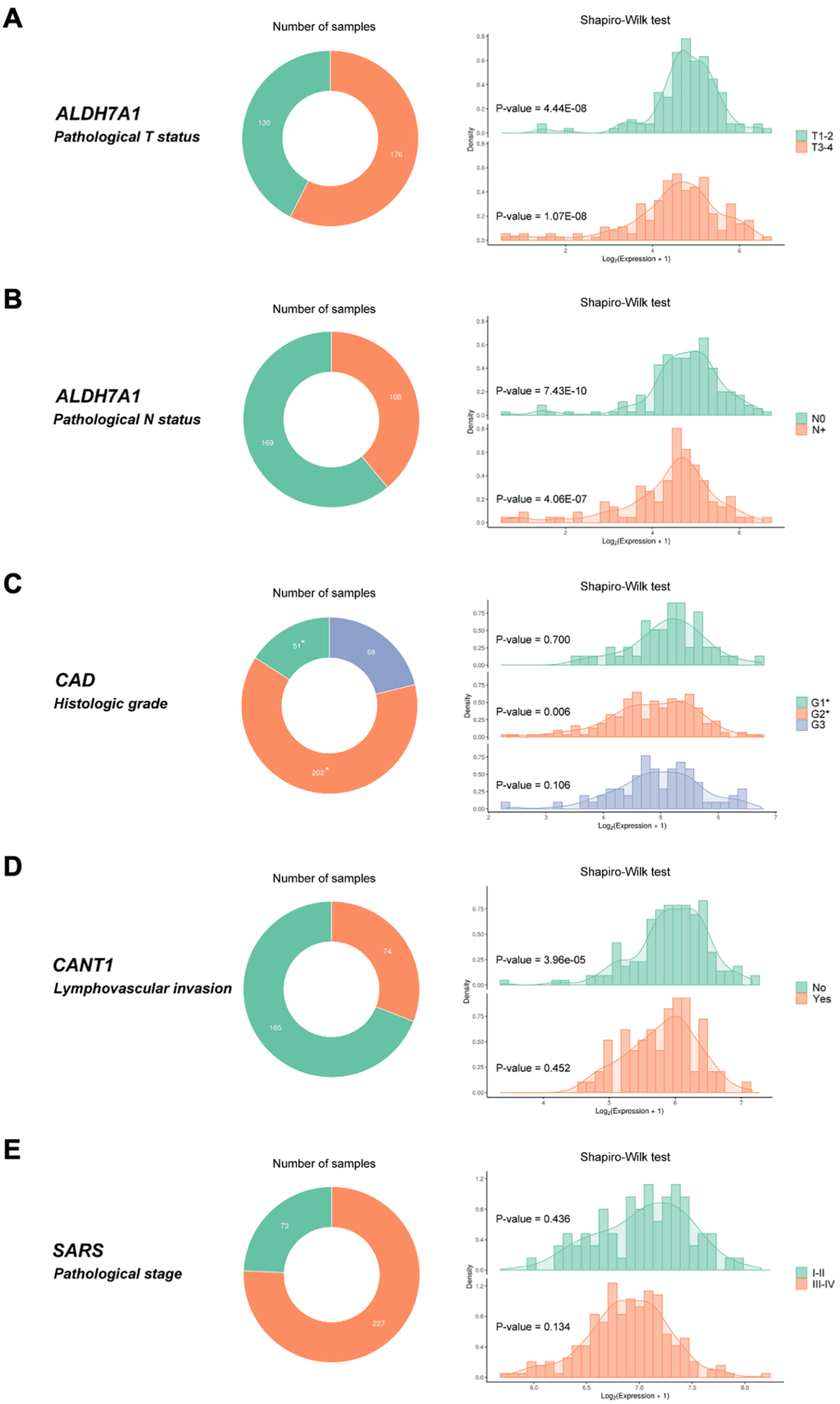
A
Number of samples
Shapiro-Wilk test
ALDH7A1
Pathological T status
130
176
P-value = 4.44E-08
P-value = 1.07E-08
Density
Log2(Expression + 1)
T1-2
T3-4
B
Number of samples
Shapiro-Wilk test
ALDH7A1
Pathological N status
108
169
P-value = 7.43E-10
P-value = 4.06E-07
Density
Log2(Expression + 1)
N0
N+
C
Number of samples
Shapiro-Wilk test
CAD
Histologic grade
51*
68
202*
P-value = 0.700
P-value = 0.006
P-value = 0.106
Density
Log2(Expression + 1)
G1*
G2*
G3
D
Number of samples
Shapiro-Wilk test
CANT1
Lymphovascular invasion
74
165
P-value = 3.96e-05
P-value = 0.452
Density
Log2(Expression + 1)
No
Yes
E
Number of samples
Shapiro-Wilk test
SARS
Pathological stage
73
227
P-value = 0.436
P-value = 0.134
Density
Log2(Expression + 1)
I-II
III-IV

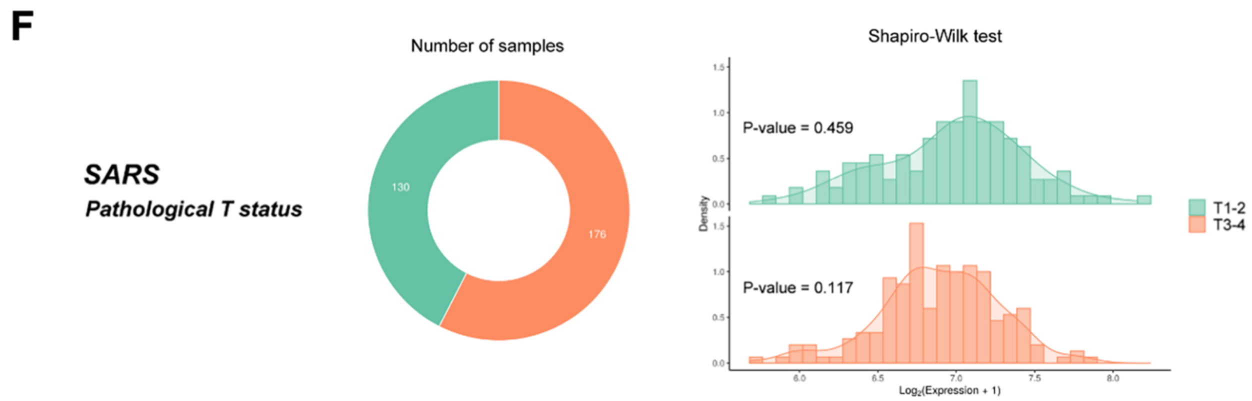

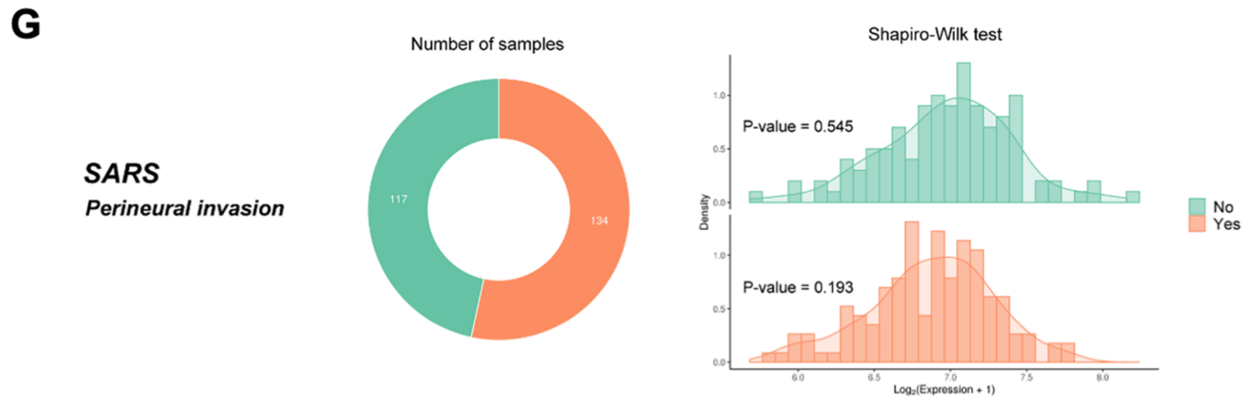

Supplemental Fig. S3. Evaluation of the normality assumption in transcript levels from hub proteins using TCGA database for OSCC. Gene expression for each clinical group was used to evaluate normality (Shapiro-Wilk test) and guide further statistical decisions to investigate the association between 'hub proteins' and prognostic features in OSCC. The number of samples (left panel, donut plot) and gene expression distribution per group (right panel, histogram) are presented for the transcripts significantly associated with clinical features *ALDH7A1* (A and B), *CAD* (C), *CANT1* (D) and *SARS* (E, F and G) (See Figure 5). P-values for Shapiro-Wilk test for each group are presented in the histograms (P-value≤0.05: not normally distributed; P-value>0.05: normally distributed). G1: grade 1, G2: grade 2, G3: grade 3; N0: patient negative for lymph node metastasis considering pathological staging; N+: patient positive for lymph node metastasis considering pathological staging. *For histologic grade and *CAD*, only G1 and G2 gene expression values were considered in statistical analysis.

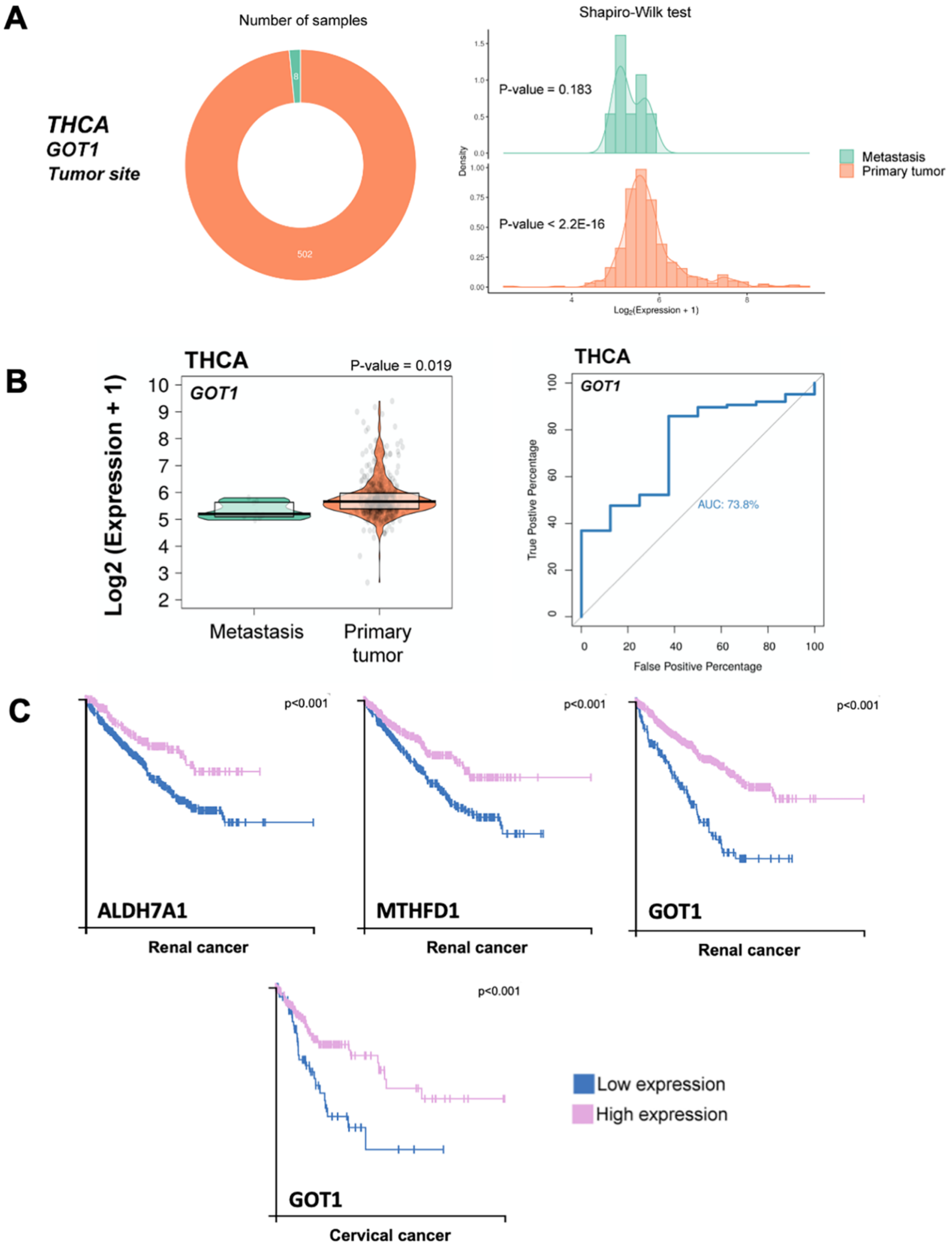

Supplemental Fig. S4. Characterization of prognostic markers in multiple tumor types. Using TCGA data, transcripts from 'hub proteins' identified in the multi-omics integrative analysis were associated with tumor site and survival in multiple cancers. The normality test (Shapiro-Wilk) considering gene expression of primary tumor and metastasis for *GOT1* in THCA is presented in (A) (left panel: number of samples; right panel: gene expression distribution and P-values for Shapiro-Wilk test per group); *GOT1* transcript levels were down-regulated in metastasis when compared to primary tumor tissues (P-value≤0.05) and could discriminate patients according to tumor location in THCA (ROC curve; AUC=73.8%) (B). Low levels of three transcripts were also associated with poor overall survival using primary tumor information from renal and cervical tumors (C) (P-value≤0.05; Human Protein Atlas platform). THCA: thyroid cancer.
